# Schwannoma with concomitant tuberculosis in the adrenal gland

**DOI:** 10.4103/0970-1591.36724

**Published:** 2007

**Authors:** Suhasini Gazula, Kim J. Mammen

**Affiliations:** Department of Urology, Christian Medical College and Hospital, Ludhiana, India

**Keywords:** Adrenal schwannoma, granulomatous inflammation, tuberculosis

## Abstract

Schwannoma is a rare primary tumor originating in the neural sheath with a good prognosis. The management is surgical. We present a case in which a retroperitoneal mass arising from the adrenal gland was excised and histopathology revealed a schwannoma with coexisting tuberculosis. This case report highlights the need to be aware of the potential coexistence of tuberculosis in any case of incidentaloma, especially due to resurgence of tuberculosis as a global epidemic with the increasing incidence of HIV and AIDS. To the best of our knowledge, this is the first reported case of a schwannoma and tuberculosis coexisting in the adrenal gland.

## INTRODUCTION

Schwannomas are usually benign tumors that arise from the nerve supporting the Schwann cell. Visceral schwannomas, in particular, juxta-adrenal and adrenal schwannomas are exceedingly rare. We report an unusual case of a schwannoma coexisting with tuberculosis in the adrenal gland, which is the first of its kind to be reported to the best of our knowledge.

## CASE REPORT

A 42-year-old Indian male presented with pain in the left flank after a trivial fall while walking. Ultrasonography of the abdomen incidentally revealed a well-defined mass arising from the right adrenal gland. The patient was not a known hypertensive and had no systemic or constitutional symptoms suggestive of tuberculosis.

General physical examination was unremarkable. On abdominal examination, a 10 × 8 cm ill-defined, non-tender, hard mass was felt in the right lumbar region.

Complete blood count, erythrocyte sedimentation rate (ESR), serum electrolytes, blood sugar, urinary catecholamines and vanillyl mandelic acid (VMA) were within normal limits. A contrast enhanced computed tomography (CECT) scan of the abdomen and pelvis revealed a mass with central necrosis and contrast enhancement arising from the right adrenal gland.

**Figure 1:**
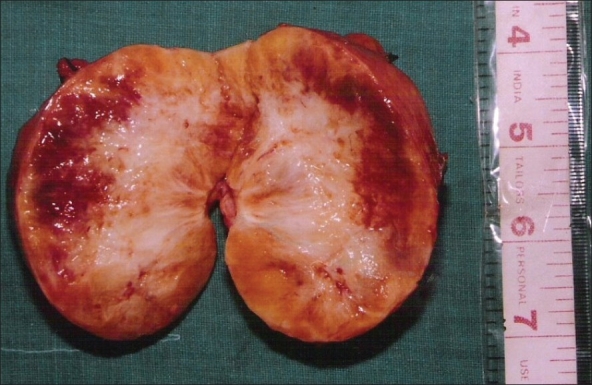
Photograph of cut section of the gross specimen of the Retroperitoneal Schwannoma showing a firm tumor with areas of haemorrhage and patchy areas of necrosis

Laparotomy was done and a well-encapsulated 12 cm × 10 cm × 4 cm tumor arising from the right adrenal gland was seen which was excised completely [Figure 1].

**Figure 2:**
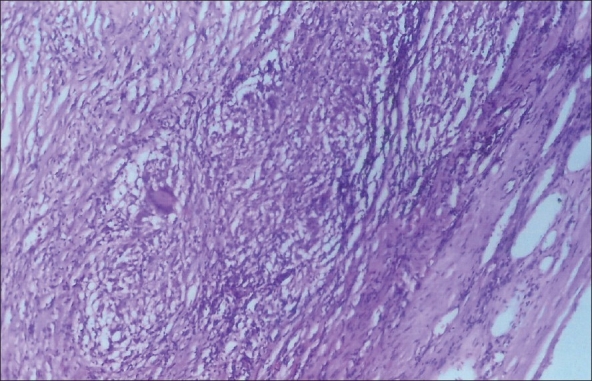
Photomicrograph of the peripheral aspect of Schwannoma showing capsule, interlacing fascicles of spindly cells and epithelioid cell granulomata. Note a Langhans Giant cell. (H&E, ×100)

Histopathological examination revealed an adrenal schwannoma with peripheral granulomatous inflammation comprising epithelioid cells and Langhans giant cells suggestive of tuberculosis [Figure 2]. Ziehl-Neelsen staining of the slides was positive. The patient was initiated on multi-drug antitubercular regimen and is well on follow-up.

## DISCUSSION

Schwannomas make up approximately 1-5% of all retroperitoneal masses.[1] They have a variable presentation and preoperative diagnosis is difficult. Although the great majority of schwannomas are benign, malignant forms also exist, frequently associated with von Recklinghausen syndrome (4% of cases) or other neurofibromatoses.[2] Benign schwannomas are not invasive but may cause the compression of adjacent structures.[1]

Most patients with retroperitoneal schwannomas present with vague abdominal or back pain. Patients are usually diagnosed in the third through fifth decades and there is an equal predilection for men and women. Laboratory studies are typically unremarkable. The CT scan usually reveals a homogeneous, solid-appearing, encapsulated mass. As schwannomas enlarge, however, they may appear cystic, secondary to hemorrhage or central necrosis.

The surgical approach should focus on complete excision of the mass. Patients undergoing complete surgical resection tend to do well without evidence of early recurrence.[3]

Tuberculous adrenal mass with no other extra-adrenal sites of tuberculosis is a very rare entity. Etiological diagnosis is usually established only after histological examination of the operative specimen.[4] With the increasing incidence of HIV and AIDS and resurgence of tuberculosis as a global epidemic, the diagnosis of adrenal tuberculosis must be considered in any case of incidentaloma,[5] particularly in the Southeast Asian region which has the highest incidence and prevalence of tuberculosis worldwide (World Health Organization, March 2007).

This case report highlights the need to be aware of the potential coexistence of tuberculosis in any case of incidentaloma, especially due to resurgence of tuberculosis as a global epidemic with the increasing incidence of HIV and AIDS.
